# Immune Response in *H. pylori*-Associated Gastritis and Gastric Cancer

**DOI:** 10.1155/2020/9342563

**Published:** 2020-01-28

**Authors:** Qingbin Niu, Jun Zhu, Xingquan Yu, Tao Feng, Hong Ji, Yuming Li, Weiwei Zhang, Baoguang Hu

**Affiliations:** ^1^Department of Gastrointestinal Surgery, Binzhou Medical University Hospital, Binzhou, Shandong Province, China; ^2^Department of General Surgery, Boxing Hospital of Traditional Chinese, Binzhou, Shandong Province, China; ^3^Department of Pathology, Binzhou Medical University Hospital, Binzhou, Shandong Province, China; ^4^Department of Anesthesiology, Binzhou Medical University Hospital, Binzhou, Shandong Province, China

## Abstract

*Helicobacter pylori* (*H. pylori*) is the dominant member of the gastric microbiota and has infected more than half of the human population, of whom 5–15% develop gastric diseases ranging from gastritis and metaplasia to gastric cancer. These diseases always follow inflammation induced by cell surface and intracellular receptors and subsequent signaling, such as the NF-*κ*B pathway and inflammasomes. Some types of immune cells are recruited to enforce an antibacterial response, which could be impeded by *H. pylori* virulence factors with or without a specific immune cell. Following decreased inflammation, neoplasm may appear with a little immune surveillance and may inhibit antitumor immunity. Therefore, the balance between *H. pylori*-associated inflammation and anti-inflammation is crucial for human health and remains to be determined. Here, we discuss multiple inflammation and immunoregulatory cells in gastritis and summarize the main immune evasion strategies employed by gastric cancer.

## 1. Introduction

*Helicobacter pylori* is a Gram-negative and motile bacterium colonizing the hostile microenvironment of the human stomach. However, *H. pylori* will continue to survive for the host's entire life, if the infections are not treated effectively. In this case, some of the patients may develop a series of diseases, including gastritis, gastroduodenal ulcer, dysplasia, and gastric cancer [1]. The main character of *H. pylori*-induced gastritis is the increased infiltration of inflammatory cells, which is usually the first detectable change among these diseases. Inflammation plays a crucial role in restricting *H. pylori*, along with the gastric mucosa damage and some immune-evasion measures to prevent *H. pylori* from immune attack. In fact, an appropriate inflammation is a feasible way to supply *H. pylori* with nutrients and decrease nutrients of the immune cells to restrict immune responses [2, 3]. Although inflammation is known for its protective role, different phenotypic immune cells may induce anti-inflammatory responses and become a fertile soil for oncogenesis. According to the immune checkpoint theory, a tumor-specific immune site attack is a cornerstone for inhibiting tumor progression to improve prognosis. Therefore, the function and regulation of immune responses in *H. pylori*-associated gastric diseases are important and ambiguous.

## 2. Invasion and Immune Response

### 2.1. Invasion and Recognition

*H. pylori* recognition and inflammation initiation may be achieved by cell surface and intracellular receptors and subsequent signaling. Pattern recognition receptor proteins (PRRs) expressed in host cells, such as Toll-like receptors (TLRs) and NOD-like receptors (NLRs), which are involved in recognizing microbial-associated molecular patterns (MAMPs), are crucial for microbial identification. TLRs, transmembrane proteins and members of the PRRs, are able to recognize the unique pathogen-associated molecular patterns (PAMPs) of *H. pylori* [4]. LPS recognition is an appropriate example of its interaction, which then induces NF-*κ*B activation and inflammatory responses. However, a lower inflammation induced via *H. pylori*-derived antigens than other microorganisms is observed.

Epithelial cells (ECs) are the first line of defense and a threshold of intracellular receptor identifying bacteria. Therefore, it is so important for *H. pylori* to transfer virulent factors into ECs. Some experiments shed light on the effect of T4SS, a syringe- and needle-like structure, which is encoded by cag pathogenicity island (cagPAI) [3, 4]. The cytotoxin-associated gene A (CagA), vacuolating cytotoxin A (VacA), and *H. pylori* cytomembrane components are easily injected into the gastroduodenal mucosa with the help of T4SS. In detail, four cagPAI proteins have been found to bind *α*5*β*1 integrin, thereby injecting CagA into the gastroduodenal mucosa [5]. In addition, CagY has a large middle repeat region (MRR) with extensive repetitive sequence motifs, and CagY MRR alternatively binds to *β*1 integrin and then attenuates T4SS function, which in turn regulates the host inflammatory response [6, 7].

The fact that *H. pylori* strains lacking a T4SS also cause inflammation suggests that other mechanisms must give hand to cytomembrane translocation. Outer membrane vesicles (OMVs) are spherical nanostructures that are shed from Gram-negative bacteria and are sufficient to trigger immune responses by *H. pylori*, although this measure is considered an alternative and takes up a small portion of invasion [8].

Nucleotide-binding oligomerization domain (NOD) is an intracellular innate immune sensor activating immune signaling pathways in response to peptidoglycans associated with microorganisms [9]. The ligand participates in NOD-1 activation via two different pathways. The first is that *H. pylori* pathogens transferred to the cytoplasm via T4SS binds with NOD-1. The other one is that outer membrane vesicles (OMVs) containing bacterial cell components (including PGN) are released by Gram-negative bacteria. NF-*κ*B is activated by NOD-2 signal to induce CXCL8 and AMPs (antimicrobial peptides) and migrate neutrophils against *H. pylori*. Other chemokines can be stimulated to recruit macrophages, dendritic cells, and Th1 cells via the NOD1-TRAF3-ISGF3 (IFN-stimulated gene factor3) pathway. Interestingly, NOD-1-activated TRAF3 prevents the expression of caudal-related homeobox 2 (Cdx2) by inhibiting NF-*κ*B activation, thereby preventing intestinal metaplasia and gastric cancer [10].

### 2.2. Inflammasome NLRP3 Activation

Multimeric protein complexes called “inflammasomes” are capable of mediating the production and formation of mature IL-1*β* and IL-18. The NF-*κ*B pathway is activated by microbial ligands and cytokines and leads to upregulation of pro-IL-1b, pro-IL-18, and NLRP3 protein levels. NLRP3 molecules form a ring-like structure, which allows the adaptor protein called apoptosis-associated speck-like protein polymerization (ASC) to recruit zymogen pro-caspase-1. The NLRP3 inflammasomes can be assembled via the recognition of PAMPs with TLRs and NOD and then activate caspase-1, which cleaves the precursors of IL-1*β* and IL-18 to generate their mature forms [11, 12]. Acid, cholesterol crystals, and other noninfectious conditions are able to promote lysosomal damage and then activate NLRP3 inflammasomes.

**Figure 1:**
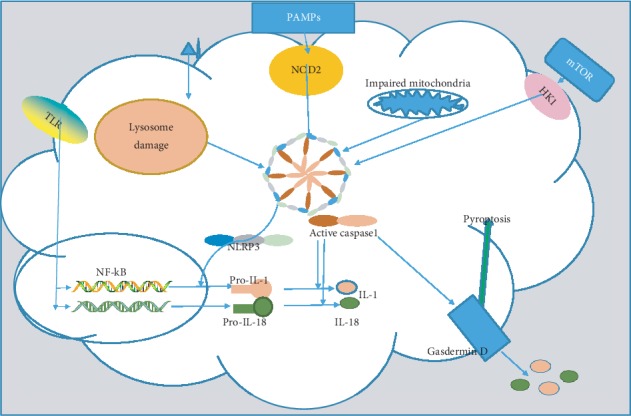
NF-*κ*B signal activation and NLRP3 inflammasome active proinflammation factors. TLR recognition induces NF-*κ*B signal activation in various pathways and then produces pro-IL-1 and pro-IL-18. NOD-2 and HK-1 receptor, impaired mitochondria, and lysosome could activate NLRP3 inflammasomes as well. Inflammasomes are capable of activating caspase-1 to cleave pro-IL-1 and pro-IL-18 into a mature molecule. Besides, gasdermin-D is assembled on the membrane to facilitate inflammation factor translocation and lead to an inflammatory cell death called pyroptosis. Abbreviations: TLR: toll-like receptors; NOD: nucleotide-binding oligomerization domain; NLRP3: NOD-, LRR-, and pyrin domain-containing 3; mTOR: mammalian target of rapamycin; HK1: hexokinase 1.

In addition, activated caspase-1 and other caspase (4/5/11) are able to cleave gasdermin-D, which exerts a role in the secretion of activated IL-1*β* and IL-18. Moreover, an inflammatory cell death called pyroptosis emerges following NLRP3 inflammasomes. Pyroptosis assists the body in restricting the growth of intracellular bacteria and exposing them to immune attacks by naive cells [11]. In addition, NLRP3 is required for the recruitment or expansion of thymus-derived and peripheral-induced regulatory T cells, which leads to increased *H. pylori* colonization [13] (Figure 1).

ECs secreting mucin-1 (MUC-1) play a key role in gastric mucosa completion and function, and MUC-1 deficiency in infected mice evokes serious gastric pathologies. MUC-1 carrying various polymorphisms is a reasonable cause to explain susceptibility to *H. pylori*-associated gastric diseases. A further study showed that *H. pylori*-infected MUC-1^−/−^Casp1^−/−^ mice did not develop severe gastritis. Both evidences indicate that inflammasomes can be regulated by MUC-1 [14].

### 2.3. Other Mechanisms That Inhibit Immunity

Gastric epithelial cells express receptors for type I (*α*/*β*) and type II (*γ*) IFN, and the subunits of these receptors are assembled in specialized cholesterol-rich membrane architecture, known as lipid rafts. *H. pylori* extract lipids from host membranes using the enzyme *γ*-glutamyl-transpeptidase (CgT), another virulence factor, mix it with its outer membrane as *α*-glucosylated derivative. Cholesterol glucosylation and extraction by *H. pylori* result in lipid raft destruction and alteration of the membrane architecture, which have been linked to IFN function deficiency. Several studies have demonstrated that although the CgT mutant loses its ability to inhibit JAK/STAT1 signaling after IFN stimulation, the CagA and cagPAI mutants still function well [15, 16]. Whether cholesterol is converted by CgT or destroyed, depletion of cholesterol leads to lipid raft destruction and then blocks the IFN signal pathway.

However, a retrospective study showed that a lot of *H. pylori*-infected individuals have been asymptomatic partly because of the inhibition of cholesterol *α*-glucoside (*α*CGL) synthesis in the cell wall of *H. pylori*, the function of 1,4-GlcNAc-terminated mucin o-glycan. In detail, invariant natural killer T (iNKT) cells can recognize *α*CGL, thereby eliciting an immune response in vitro and vivo. On the contrary, the Th1, Th17 response is reductive and the colonization of *H. pylori* is increasing with the lack of iNKT [17].

As mentioned above, epithelial integrity plays a unique role in defense, which depends on cell-cell adhesion, such as E-cadherin. Interestingly, unphosphorylated CagA-activated STAT3 not only induces temporary proinflammatory response but also disrupts cell junctions to facilitate *H. pylori* migration. Overexpressed CagA can directly interact with E-cadherin, which affects the binding of *β*-catenin to E-cadherin, resulting in *β*-catenin nuclear translocation and the Wnt signaling activation, which is usually associated with cell fate [18].

VacA prevents phagosome maturation in professional APCs. *H. pylori*-containing phagosomes fail to mature and instead resemble early endosomes (a structure with incomplete function) that have resisted lysosome fusion. This process appears to be specific to type I strains of *H. pylori*, and IFN treatment allows phagocytes to overcome their block in phagosome maturation, which kills intracellular type I strains of *H. pylori* [19].

The less expression of immunogenic ligand PAMPs is a feasible way to evade recognition by PRRs and clearance by the immune system. Thus, inhibition of MHC-II export to the cell surface and APC activation via the JAK-STAT signaling pathway may be the key point in impairing antigen presentation in dendritic cells [2, 20].

After successful phagocytosis by monocytes, *H. pylori* arginase 2 (Arg2) is able to reduce NO or O^2-^ radicals due to substrate competition responses, in other words, *H. pylori* can disrupt the NADPH oxidative system and prevent *H. pylori* from phagocytic killing [2, 21]. In addition, even though Polyamine- and NADPH-dependent generation of reactive oxygen species (ROS) is emerging, *H. pylori* prevents its damage using SOD, catalase, peroxiredoxins, and NADPH quinone reductase. Nonetheless, ROS-mediated oxidative DNA damage and mutations may participate in the adaptation of *H. pylori* to its adverse ecological niche [21].

## 3. The Different Characters of Immune Cells in Inflammation

Both innate and adaptive immunity also needs participation of immune cells, which play crucial and distinct roles in diseases. Neutrophils, macrophages, and lymphocytes exert pressure on *H. pylori* and limit its proliferation. Such strong immunity provokes intense inflammation against *H. pylori* infection, whereas a number of *H. pylori* survive in hostile environments. Interestingly, host interactions with *H. pylori* will improve allergic diseases and inflammatory bowel disease, especially in children [22]. Moreover, childhood *H. pylori* infection seems to be prevalent, which may be explained partly by reduced gastric acidity [23]. This phenomenon is significantly associated with abundant Treg cells compared to adults [24]. In fact, some kinds of regular immune cells are induced as a feasible measure to restrict inflammatory harm. However, precancerous lesion and even cancer will appear with an impaired normal immune surveillance in this process.

### 3.1. Types of T Cell

T helper (Th) cells are important types of T cells, which are thought to differentiate into CD4+ cell types with different functions under the stimulation of different cytokines. Among them, Th1 and Th17 cells are capable of eliciting an immune response against bacterial invasion [3]. In contrast, Th2 can regulate human immunity and improve a series of allergic diseases. In addition, regulatory T cell (Treg), another type of CD4+ differentiation, can suppress other effector T cells via different mechanisms [25].

Treg cells play a crucial role in immune inhibition and are regulated via the linear ubiquitin assembly complex (LUBAC). In *H. pylori*-infected subjects, suppression of the responsiveness of CD4+ memory cells has been observed, depending on the presence of *H. pylori*-specific Treg [24]. Moreover, Forkhead box P3 (FOXP3) is a key transcription factor governing Treg lineage differentiation and their immune suppressive function is encoded by the X-chromosome and affected by polyubiquitinated and deubiquitinated status. The LUBAC is composed of three proteins: ring finger protein 31 (RNF31/HOIP), RanBP-type, and C3HC4-type zinc finger containing 1 (RBCK1/HOIL-1) and SHANK-associated RH domain-interacting protein (SHARPIN/SIPL1) [26]. LUBAC plays a role in various cell signaling pathways by catalyzing and integrating linear polyubiquitin chains with substrates. It has been reported to take part in innate and adaptive immune responses after the activation of TLR, NLRP3, and NOD2. The shRNA-mediated RNF31 knockdown in human Treg cells decreases FOXP3 protein levels and increases levels of IFN-*γ*, resulting in an increase in Th1 cells. In mouse Treg cells, specific removal of RNF31 causes severe Treg cell deficiency and lethal immune diseases, revealing the importance of LUBAC activity for Treg cell homeostasis [27].

Different from the common Th cells, Th22 cells represent a newly discovered Th cell subset. The production of IL-23 by dendritic cells (DC) could polarize Th22 and then promote CXCL2 production to draw myeloid-derived suppressor cells (MDSC) during *H. pylori* infection. As an effector of Th22, MDSCs produce proinflammatory proteins, such as S100A8 and S100A9, and suppress Th1 cell responses [28].

Increased Tc17 cells, a kind of CD8+ T cells producing IL-17, is associated with tumor progression and overall survival time. The percentage of Tc17 increases with IL-6 and IL-23, and these cytokines are usually generated by tumor-activated monocytes. CXCL12 induced by supernatants cultured with Tc17 cells promotes CXCR4-dependent MDSC migration [29].

### 3.2. Monocytes/Macrophages

Monocytes/macrophages play a crucial role in inflammatory progression and tumorigenesis. The survey about absolute count of distinct white blood cells in predicting overall survival demonstrated that only lymphocytes and monocytes are capable of being independent risk factors. The combination of increased monocytes and decreased lymphocytes could further improve the predictive value for gastric cancer [30].

There are three different types of macrophages, including M1 (inflammatory macrophages), M2 (remodeling/fibrotic), and Mreg (resolving/immune-regulatory). The inherent character of M1 macrophages is proinflammatory cytokine secretion and production of nitric oxide synthase (NOS). Therefore, activated M1 macrophages compose pathogen-killing mechanisms, thereby controlling infection effectively. In contrast, activated M2 macrophages express factors of chemotactic stimuli and angiogenic properties, which are referred to as tumor-associated macrophages. In addition, the Mreg macrophages secrete high levels of anti-inflammatory cytokines, such as IL-10 and TGF-*β*. Recent studies have shown that *H. pylori* infection most often results in M1 and Mreg macrophage activation. Recruited macrophages in the site of infection can produce IL-12 that stimulates Th1 cells resulting in the production of cytokines such as IFN-*γ* [3].

### 3.3. Neutrophils

Neutrophils are the predominant leukocyte and act as the first line of host defense against invading pathogens. Proinflammatory IL17 is a critical mediator of neutrophil recruitment by CXC chemokines. Increased neutrophils are usually a powerful predictor of poor survival in gastric cancer patients [31]. Neutrophil-to-lymphocyte ratio (NLR) is correlated with clinical outcomes including overall survival (OS), progression-free survival (PFS), and objective response rate [32]. In particular, neutrophils tend to inhibit tumor growth by recruiting and activating CD8+ T cells during early phase of tumorigenesis. In addition, neutrophils can directly kill tumor cells via monoclonal antibody- (mAb-) mediated opsonization and induce tumor cell apoptosis. On the contrary, neutrophils activated by tumors prolong their lifespan and act as an immunosuppressive response on T cells [33].

However, it is still unclear which factors determine the different phenotypes of neutrophils during different tumorigenesis and tumors. A recent research identified two kinds of neutrophils including low-density neutrophils (LDNs) and high-density neutrophils (HDNs). LDNs display impaired neutrophil function and immunosuppressive properties in self-resolving inflammation and cancer, whereas HDNs possess normal neutrophil function. Incubation of whole blood from tumor-bearing mice with increasing TGF*β* indicated that increased proportion of LDNs is positively correlated with the addition of TGF-*β* dose. The proportion of LDNs significantly decreased in tumor patients via TGF*β* pathway inhibitor SB431542, whereas an obvious difference in the entire neutrophil population was not observed [34]. The neutrophils secrete matrix metalloproteinase-9 (MMP9) at cancer invasive margin, which play a crucial role in proangiogenic activity in gastric cancer [31].

### 3.4. Mast Cells

Mast cells infiltrating gastric cancer significantly increases their levels, which is a great predictor of overall survival. A study indicated that CXCL12-CXCR4 chemotaxis induce tumor-infiltrating mast cells, which express higher levels of the immunosuppressive molecule PD-L1. Mast cells induce PD-L1 expression in both time-dependent and dose-dependent manners through TNF-*α* and its NF-*κ*B signal. T cell response of inhibiting tumors could be reversed by blocking PD-L1, and the fast growth of human GC tumors is not observed [35].

Tumor-derived adrenomedullin (ADM) induced mast cell degranulation to promote proliferation and inhibit apoptosis of GC cells. This phenomenon could be achieved via the PI3K-AKT signaling pathway and reversed by blocking interleukin IL-17A production from these mast cells [36]. Another study demonstrated that increased tumor-infiltrating mast cells are correlated with angiogenesis, the number of metastatic lymph nodes, and the overall survival of patients. In detail, the release of angiogenic factors (VEGF-A, CXCL8, and MMP-9) and lymphangiogenic factors (VEGF-C and VEGF-F) is associated with the protumorigenic role of mast cells [37].

## 4. Immune Evasion by Tumor Cells

### 4.1. Immune Checkpoint Signal

#### 4.1.1. CTLA-4 Inhibits T Cell Activation Directly and Indirectly

Antigen extraction process requires a costimulus signal in addition to TCR signal and subsequent CD3 signal. One of the classic costimulations of these signals is the binding of CD28 to CD80/CD86, also known as B7, which is expressed on antigen-presenting cells (APCs). As a result of CD28 signaling, cytokine production is increased and T cell survival is promoted by induction of antiapoptotic molecular B cell lymphoma (BCL-XL) [38].

CTLA-4 (cytolytic T lymphocyte-associated antigen 4) in APCs and tumor cell inhibits T cell activation via various inhibitory pathways including competition with CD28 to produce an inhibitory signal and elevated cell motility. Binding of CTLA-4 to CD80/CD86 inhibits T cell activation via two various pathways. Specifically, binding of CTLA-4 to the B7 molecules has a higher affinity than CD28 resulting in competitive inhibition of CD28 binding to the ligand; thus, as mentioned above, phosphorylation processes are blocked by PP2A, a kind of phosphorylase. Another one is that T cell motility can also be promoted and thus lead to a relative decrease in the contact time between T cells and APC, which cannot lead to a reverse stop reaction signal and attenuates T cell activation [38].

**Figure 2:**
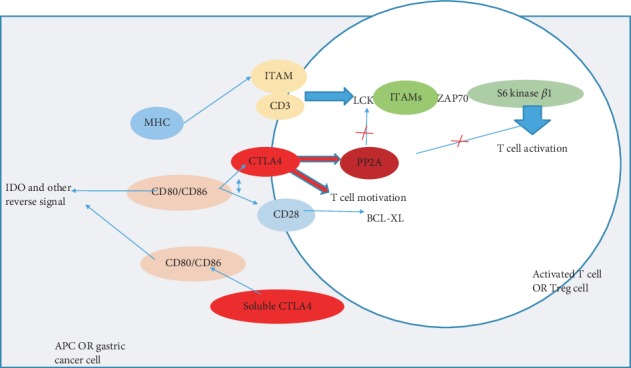
CTLA-4 inhibits T cell activation and function via CD80/CD86. In normal, CD28 combines with CD80/CD86 and delivers subsequent phosphorylation signals to maintain T cell survival. Binding of CTLA-4 to CD80/CD86 inhibits T cell activation via two various pathways. The one is CTLA-4 competition with CD28 which will block phosphorylation processes by PP2A, to disturb the normal T cell activation. Another one is that T cell motility can also be promoted and thus leads to a relative decrease in the contact time between T cells and APC and T cell activation. In addition, this signal causes some changes in APC and induces IDO. IDO has an effect on inhibiting immunity and facilitating metaplasia. Abbreviations: CD80/CD86: B7 family; CTLA-4: cytolytic T lymphocyte-associated antigen 4; LCK: lymphocyte-specific protein tyrosine kinase; ITAMs: immunotyrosine activation motifs; PP2A: protein phosphatase 2A; ZAP70: zeta-chain-associated protein of 70 kDa; IDO: indoleamine 2,3-dioxygenase.

In addition to APC blocking T cell activation by CTLA-4, the latter also has an effect on the former. Binding of the CTLA-4 molecule to the B7 family on the DCs surface seems to induce IDO expression. Indoleamine 2, 3-dioxygenase (IDO) is an enzyme that promotes the apoptosis of effector T cells by catalyzing tryptophan catabolism via the kynurenine (Kyn) pathway. IDO is involved in the differentiation of naive T cells into Treg cells. In addition, IDO is able to regulate B cell abundance during chronic gastric inflammation and produce parietal cell self-antibody to promote parietal cell loss and gastric metaplasia through ADCC [39] (Figure 2).

#### 4.1.2. PD-L and PD-1 Axis Impede Effector T Cells

PD1 signal also plays a key role in modulating T cell responses and tolerance to self-antigens. Programmed death-1 (PD-1) is a coinhibitory receptor expressed on T cells by antigenic stimulation, usually in combination with PD-L1 (also known as B7-H1) and PD-L2 (also known as B7-DC). PD-L1 expressed on Treg cells plays a role in suppressing effector T cell, and PD1 blockade is able to relieve the suppression of effector T cells. Moreover, PD-L1 expression on APCs involves the induction of peripherally derived Treg (pTreg) cells and the maintenance of pTreg cell suppressive function. Tumor-derived GM-CSF and TNF-*α* signal are able to induce PD-L1 in neutrophils and mast cells, respectively. Hypoxic condition is sufficient for inducing the expression of PD-L1 on MDSCs in the tumor context. In addition, some carcinoma can sense IFN signals and prompt PD-L1 expression via JAK-STAT. In view of the fact that tumor-derived PD-L1 can inhibit T cell function, PD1 blocking therapy is used to treat related carcinomas, whereas the effect is barely satisfactory [40].

#### 4.1.3. LAG-3 Plays an Inhibitory Role and Is Associated with Other Immune Inhibitory Receptors

Lymphocyte-activation gene 3 (LAG-3) is an immunosuppressive receptor, combining with major histocompatibility complex class II (MHC-II) as a canonical ligand. A prospective immunohistochemical analysis reported that LAG3 expression is 24.7% (21/85) and 23.6% (48/203) in gastric cancer and colorectal cancer, respectively [41]. Although LAG3 was only significantly associated with Epstein Barr virus status (*P* = 0.042) in metastatic gastric cancer, a stronger antitumor T cell response was observed following anti-LAG3 treatment [42]. Previous studies have described that LAG-3-expressing Treg cells secrete high amounts of IL-10 and TGF-*β*, both of which are capable of suppressing immune response. However, another report showed that LAG-3-deficient Treg cells did not attenuate TGF-*β* and IL-10 expression. In addition, dual blockade of the PD1 pathway and LAG3 has been shown to be more effective for antitumor immunity than blocking either molecule alone.

A puzzling phenomenon is that LAG-3 also suppresses the function of CD8+ T cells and natural killer (NK) cells facilitate the discovery of another ligand. In fact, fibrinogen-like protein 1 (FGL1) is another major functional ligand of LAG-3, which is secreted from hepatocytes and facilitates mitotic and metabolic functions of target cells. In addition, FGL1 is highly presented in human cancer cells, and elevated FGL1 in the plasma of cancer patients is associated with a poor prognosis and resistance to anti-PD-1/B7-H1 therapy [42]. The interruption of FGL1-LAG-3 interaction promotes antitumor immunity via stimulating T cell expansion and activation in the tumor microenvironment.

Soluble LAG3 (sLAG3) is a special type of LAG-3 and has a higher diagnostic value than CEA in GC. sLAG3 expression is low in peripheral blood and associated with IL-12 and TNF-*γ*. sLAG-3 might inhibit the tumor growth via promoting CD8+ T cells and secreting IL-12 and IFN-*γ* as an immune adjuvant [43].

### 4.2. Immune Inhibition by Cytokines

#### 4.2.1. Adenosine Induction in a Hypoxic Environment Obstructs Immunity by Activating A2AR and Migration

Adenosine is generally induced through CD39 and CD73 in a hypoxic environment. Hypoxia induces the release of ATP via ATP-binding cassette (ABC) transporters, pannexin 1 or connexins. Accumulated ATP has two destinations, one of which is to stimulate P2 purinergic receptors (P2XRs and P2YRs) and CD39. The other is that ATP can further be degraded to adenosine to stimulate various signal pathways, including activation of CD73 and adenosine receptors. Interestingly, approximately 60% of the Tregs from cancer patients appear to have CD39+, while only about 30% of Tregs from healthy individuals express CD39+. The difference in CD39 expression suggests that CD39 is probably a crucial molecule to affect this disease [44]. A further research demonstrated that elevated CD39 and CD73 expression relates closely to poor prognosis in patients of gastric cancer and colorectal cancer [45]. Moreover, the exosome of some tumors coexpresses CD39 and CD73. Activation of CD73 on tumor cells favors cell adhesion through epidermal growth factor receptor (EGFR) and releases matrix metalloproteinases (MMPs) that facilitate the breakdown of extracellular matrix (ECM), thus enabling tumor cells to migrate to distant organs. The activation of A2BR in tumor cells promotes proliferation and angiogenesis through secretion of vascular endothelial growth factor (VEGF). In addition, adenosine is capable of reducing transendothelial migration of effector T cells into tumors via activating monocytes.

**Figure 3:**
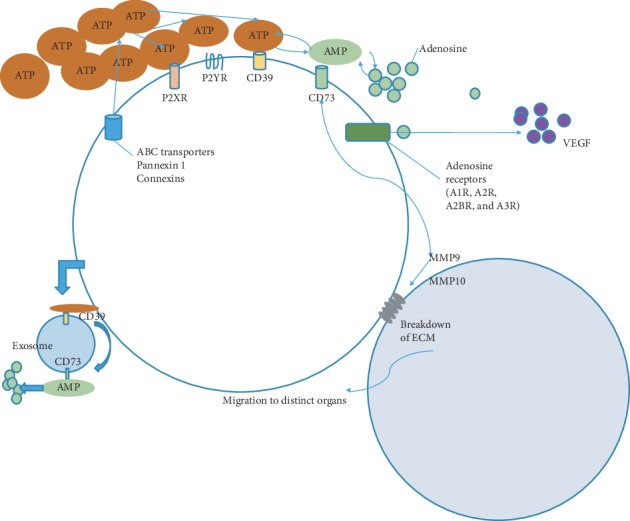
Adenosine obstructs immunity by activating A2AR and migration. Hypoxia induces the release of ATP via ATP-binding cassette (ABC) transporters, pannexin 1 or connexins. Accumulated ATP has two destinations, one of which is to stimulate P2 purinergic receptors (P2XRs and P2YRs) and CD39. The other is that ATP can further be degraded to adenosine to stimulate some various signal pathways, including activation of CD73 and adenosine receptors. The former is capable of releasing MMPs to facilitate the breakdown of ECM and tumor cell migration. The latter activation promotes tumor cell proliferation and angiogenesis through the secretion of VEGF. Moreover, the exosome of some tumor coexpresses CD39 and CD73. Abbreviations: A2AR: a kind of adenosine receptors; MMPs: releasing matrix metalloproteinases; ECM: extracellular matrix; VEGF: vascular endothelial growth factor.

In addition, IL-22 is a synergistic factor, which affects MMP-10 production of gastric epithelium via the ERK pathway. MMP-10 production is probably mediated by CagA, because CagA blocking mutants failed to increase MMP10 expression [46]. The effect of MMP-10 is paradoxical, which involves promoting gastritis and *H. pylori* proliferation. On the one hand, MMP-10-CXCL16 could be a feasible pathway for CD8+ T cell influx to prompt gastritis. However, MMP-10 can decrease Reg3a, E-cadherin, and zonula occludens-1 proteins, resulting in impaired host defense to increase Hp colonization [47]. CD39 displayed on Treg surface reduced transendothelial migration (TEM) of immune cells. When Tregs were removed from PBMCs of cancer patients, LPS-induced T cell TEM was significantly increased, while volunteers did not differ much. However, when Tregs were added back to the removed Treg fraction, a significant decrease in CD4+ T cell migration was observed. In addition, one year after the operation, transendothelial migration was restored in patients [44] (Figure 3).

## 5. Conclusion

This review discussed the immune responses in *H. pylori*-associated gastritis and gastric cancer, including *H. pylori*-induced immunity, the inflammatory regulator of *H. pylori* and host, and secondary cancer. *H. pylori* virulent factors are crucial elements of pathogenesis and immune tolerance, including CagA, VacA, and CgT. Some inflammatory factors are induced by T4SS, NLRP3, and other pathways and migrate to infected tissues to eliminate *H. pylori*. IFN, a classical inflammatory factor, plays an important role in the elimination of bacteria, and its receptors can be collapsed via *H. pylori* CgT. In addition, host atypical immune cells, such as Th22 and M2 macrophage, and other *H. pylori* factors including decreased PAMP expression, Arg, and immature phagosomes were discussed in this review.

Immune surveillance as well as inflammation is impaired in *H. pylori-*host confrontation process and cancer forms. A seemingly reasonable mechanism needs a further research to confirm it as a breakthrough to prevent cancer. Immune checkpoint theory is feasible to inhibit carcinoma and some special immune drugs used for tumor therapy. Some clinical treatment effects are not satisfactory and are improved by some adjuvants as mentioned above. In view of this, the FGL1, a LAG-3 ligand produced in the liver, which contributes to anticarcinoma immunity and albumin-globulin ratio, is a prognostic indicator. The liver physiological change for anticarcinoma immunity remains to be determined as a supplement of immune checkpoint theory. T cell is the main immune cell for anticarcinoma immunity; however, some types of T cells facilitate MDSC migration and limit immunity. Whether the MDSC infiltration hinders or improves tumor patients' overall survival is not clear. Additionally, more detailed immune cell classification analysis should be used for tumor patient prognosis. Tc17, Th22, and Treg cells suppress common effective T cell function, which may be a potential treatment direction and prognostic indicator. Different phenotypes of macrophage and neutrophils have opposite effects as well. A clear immune mechanism could be used clinically to help identify and treat individuals at risk, to better target precancerous disease and gastric cancer treatment strategies.
